# Axonal Protein Synthesis and the Regulation of Primary Afferent Function

**DOI:** 10.1002/dneu.22133

**Published:** 2013-10-01

**Authors:** Ilona Obara, Stephen P Hunt

**Affiliations:** 1 Department of Cell and Developmental Biology, University College London, Gower Street,London WC1E 6BT, United Kingdom; 2 School of Medicine, Pharmacy and Health, Durham University, Stockton-on-Tees TS17 6BH,United Kingdom

**Keywords:** local translation, the mammalian target of rapamycin complex 1 (mTORC1), pain, itch, nociceptors

## Abstract

Local protein synthesis has been demonstrated in the peripheral processes of sensory primary afferents and is thought to contribute to the maintenance of the neuron, to neuronal plasticity following injury and also to regeneration of the axon after damage to the nerve. The mammalian target of rapamycin (mTOR), a master regulator of protein synthesis, integrates a variety of cues that regulate cellular homeostasis and is thought to play a key role in coordinating the neuronal response to environmental challenges. Evidence suggests that activated mTOR is expressed by peripheral nerve fibers, principally by A-nociceptors that rapidly signal noxious stimulation to the central nervous system, but also by a subset of fibers that respond to cold and itch. Inhibition of mTOR complex 1 (mTORC1) has shown that while the acute response to noxious stimulation is unaffected, more complex aspects of pain processing including the setting up and maintenance of chronic pain states can be disrupted suggesting a route for the generation of new drugs for the control of chronic pain. Given the role of mTORC1 in cellular homeostasis, it seems that systemic changes in the physiological state of the body such as occur during illness are likely to modulate the sensitivity of peripheral sensory afferents through mTORC1 signaling pathways. © 2013 Wiley Periodicals, Inc. Develop Neurobiol 74: 269–278, 2014

## INTRODUCTION

The evidence for local protein synthesis in axons is now compelling, although our understanding of the functions supported by local translation is less complete (Zheng et al., 2001; Piper and Holt, 2004; Gumy et al., 2011; Deglincerti and Jaffrey, 2012; Jung et al., 2012). Given the length of peripheral axons, over a meter in adult humans, it was thought that local protein synthesis in axons and axon terminals must occur independently of protein synthesis in the cell body simply because of the time limits imposed by axoplasmic transport and the half-life of many axonal proteins (Alvarez and Torres, 1985). In the case of the sciatic nerve of the rat, it has been estimated that it would take several days for new proteins to reach the axon terminals in the periphery (Alvarez and Torres, 1985; Giuditta et al., 2002) and that local protein synthesis in axons would therefore be essential for maintaining axonal integrity (Koenig, 1991; Alvarez et al., 2000; Giuditta et al., 2002; Sotelo-Silveira et al., 2006). It was also argued that plastic changes that are known to occur in sensory afferents following injury could not be supported by only local post-translational changes in signaling pathways or insertion of receptor channels into the peripheral axon terminal, although the evidence for this is less clear (Giuditta et al., 2002; Bogen et al., 2012). It now seems likely that local translation of mRNA is essential for maintaining the function of most adult sensory axons and indeed is recruited to aid regeneration following nerve trauma (Zheng et al., 2001; Verma et al., 2005; Willis et al., 2005; Melemedjian et al., 2011; Obara et al., 2011a). There is also convincing *in vitro* data that local translation of mRNA can occur in developing peripheral sensory and sympathetic axons where large numbers of axonal mRNA have been identified and some mRNA directly implicated in growth cone dynamics, mitochondrial biosynthesis, and synthesis of translational machinery (Zheng et al., 2001; Cox et al., 2008; Andreassi et al., 2010; Deglincerti and Jaffrey, 2012; Kar et al., 2013;). There is also *in vitro* evidence for local translation in the axons and growing axon tips of regenerating axons (Zheng et al., 2001; Verma et al., 2005; Willis et al., 2005; Li et al., 2010; Willis et al., 2011) as well as *in vivo* data pointing to the generation of local protein signals retrogradely transported to the nucleus following axon damage (Hanz et al., 2003; Perlson et al., 2005; Huang et al., 2008; Yoo et al., 2010).

Herein, we will discuss local translation in sensory axons *in vivo* and review the evidence that local protein synthesis plays an important role in regulating the function of subsets of primary afferents, particularly nociceptors specialized to respond to damage or impending injury as well as in peripheral sensory fibers that signal itch and cooling. It is usual to associate pain with injury and to assume that once the injury has healed the pain will disappear. In fact, 16% of Europeans suffer from moderate to severe pain that does not resolve even though the injury has healed (Breivik et al., 2006). Chronic pain can be a consequence of accidents, surgery, or drug treatments like chemotherapy, and it is extremely difficult to treat with currently available drugs. It is estimated that fewer than 60% of people with chronic pain receive adequate treatment. Pain is a symptom of an underlying disease process, and it is essential to understand this disease process if rational approaches to pain control are to be developed. We work on the assumption that if we understand the underlying molecular changes in nociceptive pathways, new treatments for chronic pain can be generated. Local translation of mRNA in sensory axons is a new addition to our molecular understanding of sensory processing and has proved to be a useful new target for the control of chronic pain states.

## PERIPHERAL NOCICEPTIVE PATHWAYS

Primary afferents fall into two broad categories: myelinated A-fibers that signal noxious or innocuous stimuli and unmyelinated C-fibers that in rodents are largely nociceptors (Julius and Basbaum, 2001). A-nociceptors mediate “first” pain perceived as rapid and sharp and C-fibers signal “second” pain, delayed, diffuse, and dull. Different subsets of sensory fibers can also be characterized according to their complement of receptor proteins and neurotransmitters. The dorsal root ganglion (DRG) neurons that give rise to C-fibers can be divided into two fairly equal groups. One group of C-fibers contains and releases the neuropeptides substance P (SP) and calcitonin gene-related polypeptide (CGRP), responds to nerve growth factor through the expression of the trkA receptor and terminates within the superficial dorsal horn of the spinal cord. The second subset of C-fibers is characterized by absence of neuropeptides but the presence of the isolectin B4 (IB4) binding site and is sensitive to glial-derived neurotrophic factor (GDNF). IB4+ fibers also terminate is the superficial dorsal horn of the spinal cord but in slightly deeper laminae (Hunt and Mantyh, 2001; Todd, 2010). This distinction between subsets of C-fibers has proved to be functionally important and will be returned to later when discussing the concept of hyperalgesic priming. A-nociceptors can be divided into thinly myelinated A-delta (Aδ) fibers and larger A-beta (Aβ) fibers and most do not sensitize, in contrast to C-fibers. A-nociceptors respond to noxious heat and mechanical stimuli providing the first route for rapid signaling nociceptive information to the superficial spinal cord. Nevertheless, while both C- and some A-fibers respond to peripheral noxious stimulation subsets of fibers also signal cold and itch (Ji et al., 2007; Ringkamp et al., 2011) suggesting that there are parallel routes for sensory information to reach the dorsal horn of the spinal cord. This will prove to be significant as our analysis has identified a functional role for local protein synthesis mainly in A-fibers.

## mTORC1 AND PRIMARY AFFERENTS

**Figure 1:**
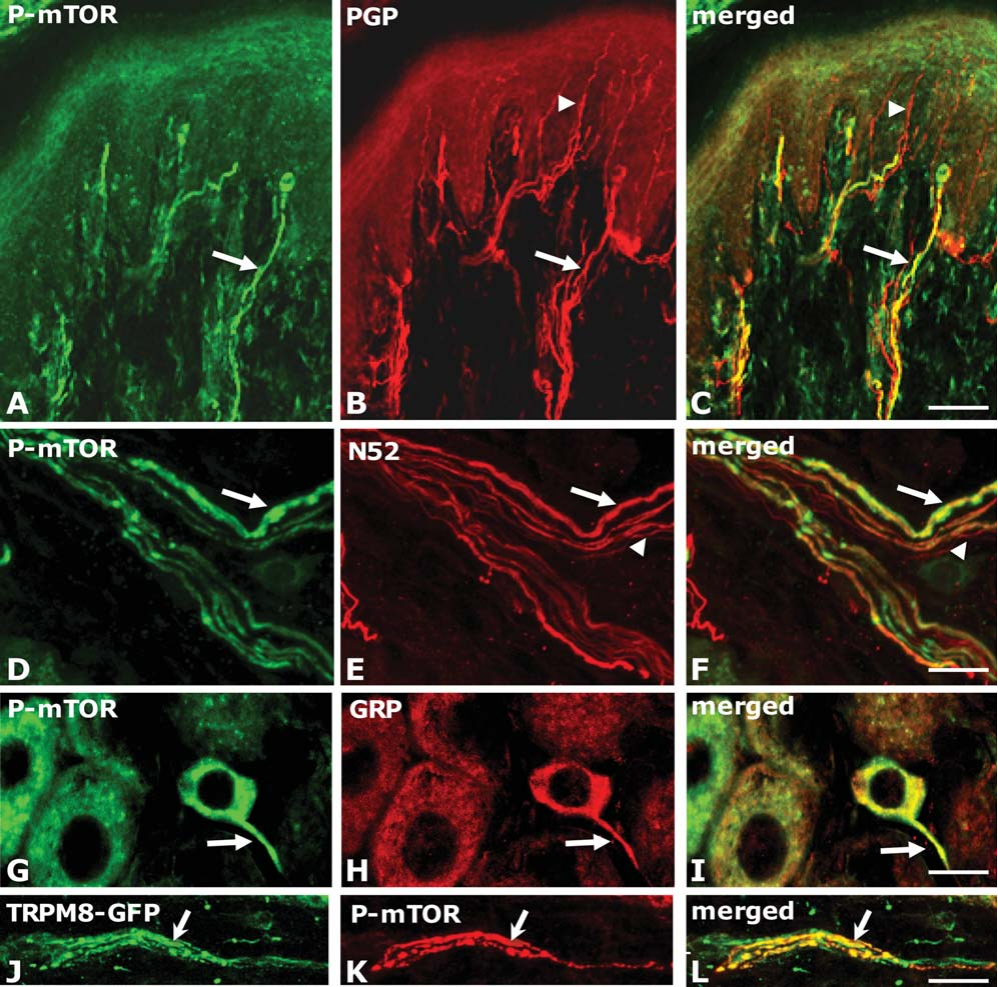
Distribution of activated mTOR (P-mTOR) immunoreactivity in peripheral sensory nerve fibers in the rodent cutaneous tissue. (A–F and J–L) Confocal images of 40 *μ*m thick frozen sections cut perpendicular to the plantar surface of the rodent hindpaw. (A–C) Arrows indicates co-localization of P-mTOR (green) and a general marker of sensory nerve fibers PGP (red) in the footpad of rat. Arrow heads indicate a fine fiber (most likely a C-fiber) entering the epidermis but not P-mTOR positive. Scale bar = 70 *μ*m. Modified from Jimenez et al. (2008). (D–F) Confocal images of a nerve bundle in the dermis co-stained with P-mTOR (green) and an A-fiber marker N52 (red). Note that all P-mTOR positive fibers are stained but that not all A-fibers are co-labeled. Scale bar = 15 *μ*m. (G–I) Confocal images of rat lumbar DRG stained with P-mTOR (green) and gastrin-releasing peptide, a marker of itch fibers (GRP, red). Note the small size of the cell body and the extension of P-mTOR immunoreactivity into the proximal axon (arrow). Scale bar = 15 *μ*m. (J–L) Confocal images of a bundle of nerve fibers in the dermis of mouse cutaneous tissue stained for P-mTOR (green) and TRPM8-GFP (red) showing some co-expression (arrow). Scale bar = 15 *μ*m. [Color figure can be viewed in the online issue, which is available at wileyonlinelibrary.com.]

Recently, it was demonstrated that a subset of sensory axons in adult rat and mouse cutaneous tissues contained an activated form the mammalian target of rapamycin, a serine/threonine protein kinase phosphorylated at serine 2448 (phospho-mTOR, P-mTOR) (Jimenez-Diaz et al., 2008; Geranton et al., 2009; Melemedjian et al., 2011; Obara et al., 2011a, 2011b; Verma et al., 2005). Using markers that distinguish C- from A-fibers, we were able to show immunohistochemically that P-mTOR+ fibers were almost exclusively A-fibers characteristically terminating within the dermis in contrast to C-fibers which often enter the overlying epidermis [Fig. 1(A–C, D–F)] (Jimenez-Diaz et al., 2008). mTOR is a master regulator of protein synthesis integrating a variety of environmental cues to regulate cellular homeostasis (Laplante and Sabatini, 2012). mTOR forms at least two multiprotein complexes known as mTOR complex 1 (mTORC1) and mTOR complex 2 (mTORC2) (Zoncu et al., 2011; Magnuson et al., 2012). mTORC1 is fairly well understood and recognized as an environmental sensor with acute sensitivity to rapamycin but far less is known about TORC2 (Zoncu et al., 2011). In a phosphorylated, active form mTORC1 can activate downstream pathways and has been shown to promote protein synthesis in cell bodies, axons, and dendrites (Costa-Mattioli et al., 2009). Ribosomal protein S6K (S6 kinase) and 4EBP (eIF4E; eukaryotic initiation factor 4E-binding protein) are well-characterized substrates for mTORC1. S6K activation absolutely requires TORC1-mediated phosphorylation and phosphorylates its own set of targets, many of which promote protein production (Magnuson et al., 2012). In a parallel pathway, TORC1-mediated phosphorylation of 4EBP1 initiates cap-dependent translation by eIF4E. Thus, TORC1 signals along parallel pathways to co-ordinately promote protein synthesis (Ekim et al., 2011; Magnuson et al., 2012).

Downstream targets of the translational machinery, including the eukaryotic initiation factor 4E-binding protein 1 (4E-BP1), p70S6 kinase (S6K), and ribosomal protein S6 (rpS6, a component of the 40S ribosome and a well-studied S6K substrate) were also found in a subset of A-nociceptors (Jimenez-Diaz et al., 2008). Ribosomes have been observed in myelinated fibers (Koenig and Martin, 1996; Kun et al., 2007; Zelena, 1970, 1972), and while some are derived from the cell body, it was recently shown that ribosomes can be transferred from myelinating Schwann cells to axons (Court et al., 2008). There is also evidence for the transport of subsets of mRNA traveling down the axon in association with RNA binding and transport proteins such as staufen and fragile X mental retardation protein (Price et al., 2006). In addition, both electronmicroscopy and in situ hybridization have revealed discrete ribosomal domains called periaxoplasmic ribosomal plaques (PARPs), distributed at periodic intervals along myelinated vertebrate peripheral axons (Koenig et al., 2000; Sotelo-Silveira et al., 2011). The weight of evidence, therefore, implies that the machinery for local translation is present in peripheral sensory fibers, particularly A-fibers.

## mTORC1 AND NOCICEPTION

**Figure 2:**
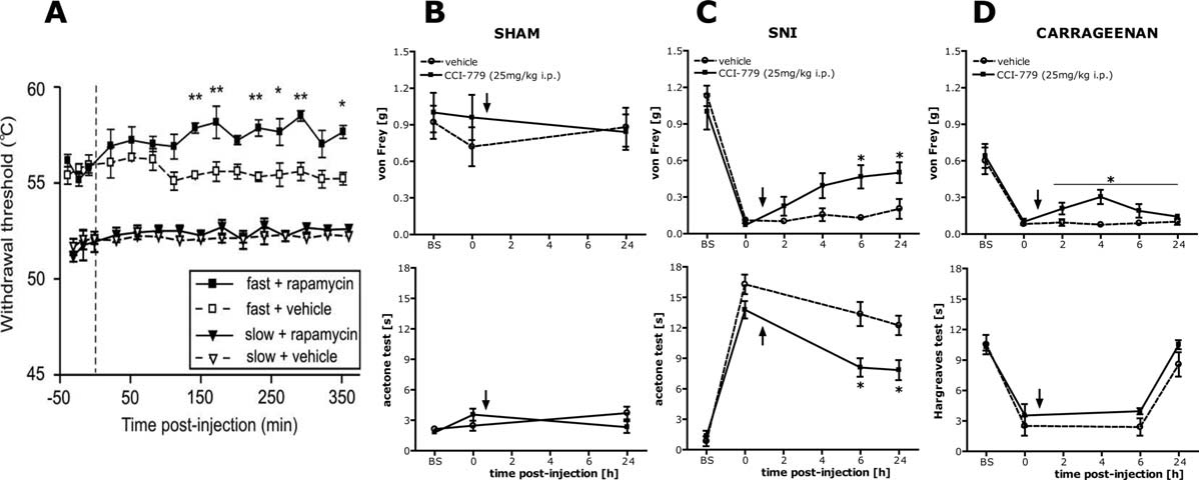
(A) Rapamycin increases A- but not C-nociceptor-evoked paw withdrawal thresholds. Time-course effects of rapamycin or appropriate vehicle, on paw withdrawal thresholds to fast and slow heat ramps that preferentially activate A- and C-nociceptors, respectively. Vertical dashed line indicates the drug injection time. *P < 0.05. Reproduced from Jimenez-Diaz et al. (2008). (B–D) In the spared nerve injury (SNI) model of neuropathic pain, intraperitoneal (i.p.) administration of rapamycin analogue CCI-779 did not affect acute pain in sham mice but attenuated mechanical (upper panel) and cold (lower panel) hypersensitivity in neuropathic mice (B–C). Note that local CCI-779 had a similar effect (Obara et al., 2011). (D) Also in carrageenan-induced inflammation i.p. injection of CCI-779 produced a transient reduction in mechanical thresholds but did not affect response to heat. Effects of i.p. injection of CCI-779 (25 mg/kg) or vehicle in sham-treated (B), SNI-treated (C), or carrageenan-treated (D) mice on the mechanical withdrawal threshold measured with von Frey filaments (B–D, upper panel), response to cold measured by withdrawal duration to the acetone drop test (B-C, lower panel), and withdrawal latency to heat measured by the Hargreaves test (D, lower panel). The measurements were assessed before injury as basal pain threshold (BS) and then 3 days (sham and SNI) or 12 h (carrageenan) after the injury (time point 0). The effect of CCI-779 was assessed 2, 4, 6, and 24 h after i.p. injection. Arrows indicate CCI-779/vehicle injection time. *P < 0.05. This figure has been reproduced with permission of the International Association for the Study of Pain® (IASP). The figure may NOT be reproduced for any other purpose without permission.

*mTORC1 and A-nociceptors*. The functional role of activated mTORC1 in sensory fibers was studied in two ways. First, electromyography was used to separate the responses of A- and C-nociceptors (Jimenez-Diaz et al., 2008). Heat responsive and capsaicin-insensitive A-nociceptors located within the dorsal hairy skin of the hindpaw were preferentially activated by a fast heat ramp applied to the hind paw, whereas C-fibers responded only to a slower heat ramp. Subcutaneous injection of rapamycin into the dorsal skin of the hindpaw significantly increased threshold temperatures for paw withdrawal evoked by fast heat ramps that activated A-fiber nociceptors but did not change thresholds of C-fibers activated by slow heat ramps [Fig. 2(A)]. In addition, extracellular compound action potentials were recorded from dorsal roots in vitro (Geranton et al., 2009). Rapamycin treatment of isolated dorsal roots significantly increased the threshold stimulation intensity required to activate the Aδ component compared to vehicle-treated roots. In contrast, there was no significant difference in the threshold values for activation of the A-alpha (Aα), Aβ, or the C-fiber components. The conduction velocities and amplitudes were also not significantly altered by rapamycin treatment. A second piece of evidence suggesting that A-fibers were the peripheral targets of rapamycin was the demonstration of an attenuated second phase of behavioral response following local injection of formalin which activates both A- and C-fibers (Asante et al., 2009; Price et al., 2007). Capsaicin, the active “hot” component of chilli pepper specifically activates C-nociceptors but the effects of capsaicin on C-fibers were not modified by local rapamycin injection (Jimenez-Diaz et al., 2008) suggesting that the formalin deficit was largely due to an inhibition of A-nociceptor function. The same effect was found after subcutaneous injection of anisomycin when compared with vehicle (Jimenez-Diaz et al., 2008). This indicated that A-nociceptor function alone could be selectively reduced by mTORC1 inhibition.

The effect of rapamycin on a subpopulation on capsaicin insensitive A-nociceptors was particularly significant because a unique role had previously been demonstrated for these sensory afferents in humans (Magerl et al., 2001). Injury results in peripheral sensitization of C-fibers not requiring local protein synthesis. Injury also results in a barrage of C-fiber nociceptive impulses into the dorsal horn of the spinal cord generating central sensitization, effectively a second level of signal amplification. Central sensitization is the result of a complex series of changes in neuronal excitability driven by descending pathways from the brainstem, local interneurons, and glial activity (Kuner, 2010). The end product of this process is that A-fiber signals arising from tissue around the site of injury are amplified within the dorsal horn of the spinal cord. In humans, this is felt as an increase in mechanical sensitivity in tissue surrounding the peripheral injury and again provides an early warning signal during the healing of vulnerable tissue. Generally, in humans, there is both an increased punctate or pinprick mechanical sensitivity (also known as secondary hyperalgesia), and in some cases, light touch can also feel aversive (allodynia). This increased mechanical sensitivity is mediated by capsaicin-insensitive A-fibers and reflects central sensitization within the central nervous system *not* peripheral sensitization (Magerl et al., 2001). We, therefore, examined the effect of rapamycin on punctate mechanical hyperalgesia in rat. We first induced central sensitization with an injection of capsaicin into the central part of plantar surface of the hind paw. Following this, we tested the punctate mechanical sensitivity that develops around the site of injection and found that it was blocked by rapamycin. Importantly, the initial peripheral sensitization by capsaicin was unaffected (Jimenez-Diaz et al., 2008).

Thus, a subset of P-mTOR containing A-nociceptive primary afferents supported the increased punctate mechanical sensitivity that develops around a site of injury but did not influence peripheral sensitization or indeed the acute pain responses.

*mTORC1 and C-nociceptors*. One essential characteristic of most C-nociceptors is that they sensitize following injury resulting in a reduction in their activation threshold and an increase in the magnitude of the response to noxious stimulation (Gold and Gebhart, 2010). For example, inflammation or tissue injury provokes the release of a variety of cytokines (i.e., interleukin-6, IL-6) and growth factors (i.e., nerve growth factor, NGF) that act on and increase the sensitivity of a subset of C-nociceptors to both noxious and non-noxious stimulation, resulting in primary hyperalgesia. The function of this increased sensitivity is to heighten awareness of the damaged tissue and avoid further damage during the healing process. This increased peripheral sensitivity to thermal and mechanical stimuli is familiar to most of us. For example, it is commonly associated with sunburn, and mechanistically is thought to be entirely the result of local post-translational changes in kinases notably p38 MAPK and ion channels such as the vallinoid receptor type I (TRPV1) in the terminal processes of nociceptors within inflamed cutaneous tissue (Ji et al., 2002). These changes are backed up at later time points by proteins synthesized in cell bodies in the dorsal root ganglia and transported to the axon terminals in inflamed cutaneous tissue (Ji et al., 2002). This process is not however thought to involve local translation of mRNA although there have been reports of mRNA for TRPV1 in the processes of sensory neurons (Tohda et al., 2001). In addition, it was also observed that the rapid sensitization of cutaneous C-fibers expressing the TRPV1 receptors produced by local injection of capsaicin, a TRPV1 agonist, was not prevented by mTORC1 inhibitor rapamycin or the broad-spectrum translational inhibitor anisomycin (Jimenez-Diaz et al., 2008; Obara et al., 2011b). Also, local injections of rapamycin and cordycepin were reported to have no effect on the mechanical hyperalgesia associated with inflammation of the paw generated by complete Freunds adjuvant (CFA) (Obara et al., 2011b; Ferrari et al., 2013), although a reduction in the mechanical and thermal hyperalgesia that followed carrageenan-mediated inflammation of the paw was seen following systemic or intrathecal rapamycin treatment [Fig. 2(D)] (Norsted Gregory et al., 2010; Obara et al., 2011b). This may have been due to a direct effect of the drug on the central nervous system. Taken together, the data suggest that the sensitization of C-fibers does not require mTORC1-mediated signaling.

*Is there a role for mTORC1 in the regulation of C-fiber function?* While there is a considerable amount of evidence that activated mTOR regulates A-fiber function particularly in nociception there is also some evidence that C-fiber excitability can be regulated by rapamycin or similar drugs. First, when direct recordings were made from teased peripheral nerve fibers in a skin-nerve preparation after the application of rapamycin or control solution, rapamycin increased the mean mechanical threshold for A-fibers *and* C-fibers by 58% and 54%, respectively, indicating that rapamycin had an effect on subpopulations of both C- and A-nociceptive fibers (Jimenez-Diaz et al., 2008). This was unexpected but does suggest that C-fibers may have some capacity for mTORC1 mediated local translation even though we did not find convincing evidence for activated mTOR in C-fibers using immunohistochemistry. Second, in a recent study, mTORC1 was also found to be essential for hyperalgesic priming defined as “a long lasting latent hyper-responsiveness of nociceptors to inflammatory mediators or neuropathic insult” (Bogen et al., 2012). This was thought to be mediated largely by C-fibers and considered by the authors as a model of transition from an acute to chronic pain state (Ferrari et al., 2013). It was shown that the IB4+ group of C-fibers was essential to the development of a second longer lasting hyperalgesic response following an initial injury (Joseph and Levine, 2010; Bogen et al., 2012). Priming required activation of protein kinase C epsilon (PKCε) in IB4+ C-fibers that laid down a molecular memory generated by cytoplasmic polyadenylation element binding protein (CPEB) an RNA-binding protein that recruits the necessary molecular components to translate mRNA dormant in the axonal cytoplasm (Bogen et al., 2012). The target mRNA were not identified but presumed to increase neurotransmitter release at the primary afferent terminal within the dorsal horn. Recently, the same group has shown that priming following an initial carrageenan injection into the paw followed by an injection of prostaglandin E2 (PGE2) 4–7 days later is blocked by local rapamycin or cordycepin injections into the inflamed paw just before or at the same time as the initial carrageenan inflammation (Ferrari et al., 2013). These results suggest a predominant role for mTOR in IB4+ C-fibers in priming and perhaps therefore the move from acute to chronic pain states. Nevertheless, IB4+ axons in cutaneous tissues penetrate the epidermis, and we were only able to detect P-mTOR in sensory afferents in dermal tissues [Fig. 1(A–C)]. It may well be that other activated forms of mTOR are present in C-fibers but this remains to be explored (Ekim et al., 2011). However, a role for PGE2 in heat hyperpathia mediated by A-delta fibers has also been reported and suggests that activated mTOR in A-fibers may contribute to hyperalgesic priming (Bastos and Tonussi, 2010). Finally, IL-6 injected into the mouse hindpaw has been shown to generate a rapamycin-reversible mechanical hypersensitivity (Melemedjian et al., 2010; Price and Geranton, 2009). Given the widespread distribution of IL-6 receptors on DRG neurons and the complex role that IL-6 plays in the regulation of nociception this result may be the result of a combined effect of IL-6 on both C- and A-nociceptors (Murphy et al., 1995, 1999). Finally, it is also possible that local protein synthesis in C-fibers may be under the control of signaling molecules other than mTOR, and there is evidence that extracellular regulated kinase (ERK) may modulate local translation of mRNA in C-fibers (Price and Geranton, 2009).

*mTORC1 and neuropathic pain*. A major step in the functional analysis of local translation in sensory fibers was the recognition that the capsaicin-insensitive A-fibers formed a distinct functional group of fibers both in man and rodents (Magerl et al., 2001; Jimenez-Diaz et al., 2008). The importance of these A-nociceptors can be summarized as follows. First, the increased behavioral response (mechanical hyperalgesia and allodynia) to stimulation of these fibers acts as a measure or barometer of central sensitization within the central nervous system, and second, these A-nociceptors do not sensitize but their basal excitability can be reduced by rapamycin. This is important because maintained central sensitization is thought to be characteristic of many chronic pain states where light touch or moderately painful stimulation can cause excruciating pain (Schmelz, 2009). It follows then that inhibiting the function of A-nociceptors should reduce the stimulation-induced mechanical hypersensitivity associated with neuropathic pain. In rodents, partial peripheral nerve lesions are commonly used to generate models of neuropathic pain in animals. We, therefore, extended our analysis to demonstrate that rapamycin treatment would alleviate mechanical hypersensitivity found in the spared nerve injury (SNI) model of neuropathic pain [Fig. 2(B,C top)]. Local or systemic injections of rapamycin or the analogue temsirolimus (CCI-779) or the ATP-competitive inhibitor Torin 1 resulted in a significant reduction in the mechanical sensitivity associated with nerve damage (Jimenez-Diaz et al., 2008; Obara et al., 2011b). The implication of these experiments was that local injection of rapamycin or the analogue CCI-779 was able to reduce the mechanical sensitivity of A-nociceptors and so control neuropathic pain. Recent studies have broadened the significance of mTORC1 inhibition as a means of controlling chronic pain by showing that the drug metformin, widely given to control type 2 diabetes, also leads to the inhibition of mTOR and a reduction in translation initiation (Dowling et al., 2007). Systemic metformin has been shown to be effective in reducing neuropathic pain in rats and mice (Melemedjian et al., 2011, 2013). It should be noted here that the possibility that rapamycin is damping down a local inflammatory response in the paw, and so reducing inflammatory sensitivity is unlikely as the mechanical sensitivity in nerve injury models arises from the induction of central sensitization and the amplification of A-fiber responses-not local inflammation. Also, as mentioned above, rapamycin has little effect on inflammation-induced primary sensitization.

## mTORC1 AND COLD RESPONSE

Having established the contribution of activated mTOR to A-nociceptor function, we questioned whether other modalities, in part signaled by A-fibers, might also be compromised by local or systemic rapamycin. These include responses to cold and to itch. In the rodent model of neuropathic pain, there is a heightened response to cold stimuli usually generated by evaporation of a drop of acetone to the paw [Fig. 2(B,C) bottom]. This response to cold was attenuated by rapamycin or CCI-779 (Obara et al., 2011b). There is good evidence that the signaling of cool temperatures that become aversive in neuropathic pain models is through the TRPM8 receptor a member of the transient receptor potential (TRP) channel family. TRPM8 receptor is activated by temperatures around 18°C and is sensitive to menthol and acetone-induced cooling (Dhaka et al., 2007, 2008; Knowlton et al., 2013). In TRPM8 knockout mice, there was a reduced response to acetone-induced evaporative cooling of the hind paw in mice with the spared nerve injury model of neuropathic pain, and a reduced effect of cold was also found in inflammatory pain models (Dhaka et al., 2007; Knowlton et al., 2013). How might TRM8 sensitivity be reduced by rapamycin treatment locally? We examined the distribution of TRPM8 in naïve mice in which green fluorescent protein (GFP) had been “knocked in” to the TRPM8 locus (Dhaka et al., 2008). Using immunohistochemistry (IHC), we found occasional axons double-labeled with activated mTOR and GFP in mouse cutaneous tissues [Fig. 1(J–L)], but accounting for less than 4% of positive p-mTOR fibers (n = 2; 50–70 fibers/animal) (unpublished observations). Although cool-sensitive fibers are largely C-fibers, this lead us to suspect that the small number of P-mTOR positive A-fibers we detected are probably sensitive to cooling and contribute to the acetone sensitivity in nerve injury models of neuropathic pain. In mice, the numbers of TRPM8 positive Aδ-fibers (but not C-fibers) were reported to increase after nerve injury (Ji et al., 2007). We, therefore, concluded that a small number of TRPM8 cool-sensitive fibers are likely to be P-mTOR positive A-fibers.

## mTORC1 AND ITCH SIGNALING

Recent research has emphasized the role of C-fibers in pruritus research, but there is evidence that some A-fibers are also sensitive to the two classes of histaminergic and nonhistaminergic stimuli widely used to stimulate itch (Schmelz, 2010; Ringkamp et al., 2011; Namer and Reeh, 2013). Indeed, it was found that itch sensation was reduced during A-fiber block and recent studies confirmed the contribution of A-fiber signaling to both histamine-mediated and histamine-independent itch sensation. We, therefore, examined the co-localization of activated mTOR with gastrin-releasing peptide (GRP), a marker for some itch-sensitive primary afferents (Sun and Chen, 2007; Sun et al., 2009). GRP-positive neurons in the dorsal root ganglia of rat are extremely small in diameter but occasional neurons could be found that co-expressed GRP and P-mTOR in the initial axon segment [Fig. 1(G–I)]. In mice, we also found evidence that P-mTOR was co-expressed in less than 5% of GRP-positive fibers in cutaneous tissue and behaviorally that the itch response to histamine or nonhistaminergic pruritogens were inhibited by local and systemic CCI-779 (unpublished data). It seems likely that the reduction of the itch response was caused by inhibition of mTORC1 within specific subsets of presumed A-fiber fibers known to contribute to both nonhistaminergic and histaminergic itch.

## CONCLUSIONS: mTORC1 AND HOMEOSTASIS

mTORC1 regulates the response of A-fibers to noxious stimulation as well as to itch-provoking stimuli and cooling. Yet, the mRNA targets of translation remain unknown. Axons contain many thousands of mRNA that encode for proteins involved in most cellular functions (Deglincerti and Jaffrey, 2012). Abundant axonal mRNA transcripts encode mitochondrial proteins, and a recent in vitro study found that selective inhibition of axonal protein synthesis has been shown to reduce axonal mitochondrial membrane potential perhaps contributing to a reduced axonal membrane potential (Hillefors et al., 2007; Aschrafi et al., 2008). mRNA transcripts encoding ribosomal proteins are also highly represented and transcripts for most ribosomal proteins can be found in axons as well as mRNA coding for the translational machinery. Thus, a significant portion of local translation in axons is likely to be dedicated to maintaining an adequate supply of ribosomal proteins and the integrity of the axon. RNAs encoding eukaryotic translation initiation factors eIF2B2 and eIF4G2 are present in the axons of rat sympathetic neurons in vitro and are locally translated under the control of noncoding microRNA (Kar et al., 2013).

Regardless of how function is regulated, it might also be worth asking why P-mTOR is present in such a selective group of sensory afferents. Craig (2002) suggested that pain, temperature, and itch sensations should be distinguished from touch and represent signals from the body that are “directly related to homeostatic needs” and regulate the internal milieu of the animal. mTORC1 is known to play a crucial role in the signaling pathway that regulates cell homeostasis in response to a variety of external stressors and cues including nutrients and growth factors, hypoxia, DNA damage, mechanical damage, and osmotic stress (Reiling and Sabatini, 2006; Zoncu et al., 2011), and it seems likely that the complex peripheral changes in the physiological state of the body, for example during illness, may be reflected in modulation of the sensitivity of peripheral sensory afferents by mTOR signaling pathways.
